# Association of School District Policies for Radon Testing and Radon-Resistant New Construction Practices with Indoor Radon Zones

**DOI:** 10.3390/ijerph13121234

**Published:** 2016-12-13

**Authors:** Stephanie Foster, Sherry Everett Jones

**Affiliations:** 1 Agency for Toxic Substances and Disease Registry, Atlanta, GA 30341, USA; 2 Centers for Disease Control and Prevention, Atlanta, GA 30329, USA; severettjones@cdc.gov

**Keywords:** radon, schools, school district, testing, radon-resistant new construction, policy

## Abstract

Radon is a naturally occurring, colorless, odorless, and tasteless radioactive gas. Without testing, its presence is unknown. Using nationally representative data from the 2012 School Health Policies and Practices Study, we examined whether the prevalence of school district policies for radon testing and for radon-resistant new construction practices varied by district location in relation to the U.S. Environmental Protection Agency Map of Radon Zones. Among school districts located in counties with high predicted average indoor radon, 42.4% had policies for radon testing and 37.5% had policies for radon-resistant new construction practices. These findings suggest a critical need for improved awareness among policy makers regarding potential radon exposure for both students and school staff.

## 1. Introduction

Radon gas is the result of the natural decay of uranium found in the Earth’s underlying bedrock [1,2,3]. According to the United States Environmental Protection Agency (EPA) radon is, “one of the most hazardous indoor pollutants” and is “the leading cause of lung cancer among non-smokers” contributing to an estimated 21,000 lung cancer deaths each year [2]. Testing is the only way to know of radon’s presence because it is colorless, odorless, and tasteless. Short-term, typically 2–90 days, and long-term, greater than 90 days, tests are available. The short-term tests are simple and relatively inexpensive and guidance for testing is freely available from the EPA [4].

Mandated by Congress via the Indoor Radon Abatement Act of 1988, EPA was charged with the responsibility of identifying areas of the United States with potentially elevated indoor radon levels [5]. Using information from a combination of geologic, aerial radioactivity, soil characteristic measures, indoor radon measurements, and building foundation type, EPA created the Map of Radon Zones [6]. This map classifies counties into one of three zones depending upon the levels of predicted average indoor radon. Because high indoor radon levels can be found in all three zones, EPA asserts that all buildings should be tested regardless of geographic location [6].

When testing reveals levels above the EPA’s recommended action level of 4 picocuries per liter (pCi/L), several mitigation strategies are available for reducing the indoor radon levels [7,8]. Although the proper system is dependent upon the building, vent pipe systems with fans are often used to pull radon from the area beneath a building and vent it to the outside. EPA’s publication, Radon Prevention in the Design and Construction of Schools and Other Large Buildings, describes in detail mitigation methods [9]. Additionally, the EPA emphasizes the importance of considering radon prevention features during the building design process. Incorporating radon-resistant features during building construction “…makes their application easier and costs much less than adding them after the building is completed” [9].

Because children’s bodies are still developing, they are susceptible to hazardous substances from environmental exposures, including radon [1]. As such, children are vulnerable to environmental exposures at school because of the many hours they are required to spend in school. The findings of a nationally representative survey conducted in the 1990s by the EPA suggest evidence of “widespread radon contamination in schools” [4]. That study estimated nearly one in five schools, approximately 19% of U.S. schools, and more than 70,000 schoolrooms had indoor radon levels greater than 4 pCi/L in at least one frequently occupied ground contact room [4].

Some states participate in active radon testing and mitigation programs. States like Colorado, Connecticut, Florida, Illinois, and Virginia have a long history of legislation regarding radon testing. For example, between 2000 and 2007 New Jersey tested 1705 (51%) public schools [10]. However, overall, laws and regulations for reducing radon in U.S. schools are scarce [11]. Additionally, historical review of laws and regulations uncovers fluctuations over time in school radon testing and radon-resistant new construction policies [12,13,14,15].

Given the potential widespread occurrence of radon in schools we were interested in evaluating the relationship between reported policies for radon testing and radon-resistant new construction and indoor radon potential. Therefore, the purpose of this study was to examine whether the prevalence of school district policies for radon testing and for radon-resistant new construction practices differed across EPA’s three radon zones.

## 2. Materials and Methods

### 2.1. School Health Policies and Practices Study 2012

The School Health Policies and Practices Study (SHPPS) 2012 was conducted by the Centers for Disease Control and Prevention (CDC) during October 2011–August 2012 [16]. SHPPS 2012 data assess the characteristics of school health identified in the Whole School, Whole Community, Whole Child model [17]. This analysis examined data from the SHPPS district-level Healthy and Safe School Environment questionnaire which included questions about policies for radon testing in schools and radon-resistant new construction practices for new school campuses or renovations.

A detailed description of the SHPPS 2012 methods has been published previously [18]. SHPPS 2006 questionnaires underwent a question-by-question review to determine questionnaire content for 2012. New questions added for SHPPS 2012 and questions that were modified substantially from SHPPS 2006 were subjected to cognitive testing using telephone interviews. Draft questionnaires were evaluated by reviewers from federal agencies, national associations, foundations, universities, and businesses nationwide and appropriate revisions were made. Sampled districts were asked to identify respondents who were responsible for, or most knowledgeable about, the component covered within a questionnaire or module. SHPPS 2012 was reviewed by the Institutional Review Boards at both CDC and ICF Macro, Inc. (Rockville, MD, USA), an ICF International Company (contractor engaged for SHPPS 2012) and determined to be exempt.

A nationally representative sample of public school districts was invited to participate. Primary sampling units (PSUs) were defined broadly as groupings of contiguous school districts. PSUs were constructed to facilitate weighting and variance estimation so that an equal probability sample was achieved. The PSUs were classified into four strata defined by urbanicity (urban or non-urban) and socioeconomic status (high-poverty or low-poverty). PSUs were sampled with equal probability without replacement. All districts in the sampled PSUs were included in the sample. In addition to these sampled PSUs, 20 certainty PSUs were added to the sample. These PSUs were the 20 districts funded by the CDC at the time.

Most (85.4%) of the district-level questionnaires were completed via web-based self-administration; the remaining 14.6% were completed using self-administered paper and pencil questionnaires. The Healthy and Safe School Environment questionnaire was comprised of 4 modules that grouped related items so a single respondent could complete each module, and allowed for different respondents for each module as appropriate. The response rate for the module containing the physical school environment questions was 57.1% (598/1048).

### 2.2. Measures

SHPPS 2012 included two district-level questions related to radon: whether the district had adopted a policy requiring that schools be tested for radon and whether the district had adopted a policy addressing the use of radon-resistant new construction practices for new school campuses or renovations (response options were “yes” or “no” for both questions). An analysis of districts with missing data for those policy questions (*n* = 137 and *n* = 141, respectively) found the distribution of districts in EPA radon Zone 1, 2, or 3 was similar to the distribution of districts without missing data for those policy questions.

United States county radon zone data are publically available [19]. Indoor radon potential is characterized into three categories relative to the EPA’s action level of 4 pCi/L. Zone 1 counties are areas having predicted average indoor levels above the action level (>4 pCi/L), Zone 2 counties have predicted average indoor radon between 2 and 4 pCi/L, and Zone 3 counties have the lowest predicted average indoor radon (<2 pCi/L) [6].

SHPPS data were linked with extant data from the Market Data Retrieval (MDR) database (now MCH Strategic Data, Sweet Springs, MO, USA). The MDR database is updated annually and contains information about individual U.S. school districts. We used the school district locations that resulted from linking MDR data with SHPPS data. The latitude and longitude data from SHPPS were transformed into points and mapped using Esri ArcGIS 10.2 (Esri, Redlands, CA, USA). Extant data from Google Maps were linked to SHPPS data to verify both the zip code and the latitude and longitude of the school district office. All school district offices were assigned a radon zone category by the spatial joining of latitude and longitude points to county boundaries from Esri’s detail county boundary shapefiles and then linking these data to EPA radon zone designations.

### 2.3. Analysis

District level data were weighted to produce national estimates. The base district weight, or sampling weight, was computed as the inverse of the selection probability. Base weights were adjusted for nonresponse using a simple ratio adjustment, computed as the ratio of weighted totals within weight adjustment classes. Because response rates were calculated for each questionnaire, the weight for nonresponse was calculated separately by questionnaire, resulting in a set of questionnaire-specific weights for each district to be used for questionnaire-specific analyses. This analysis was conducted in SUDAAN statistical software (RTI International, Piedmont, NC, USA) which accounted for the sampling design and weighting of the data at the district level. Chi-square tests were used to examine whether the prevalence of school district policies requiring schools be tested for radon and addressing radon-resistant new construction practices differed across EPA’s three radon zones. A *p*-value < 0.05 was considered statistically significant.

## 3. Results

Overall 37.0% of districts adopted a policy requiring schools test for radon and 33.4% adopted a policy addressing the use of radon-resistant new construction practices for new school campuses or renovations. There is a statistically significant difference in the percentage of districts with a policy requiring schools be tested for radon across EPA radon zones (χ^2^ = 3.9, *p* = 0.02): highest among those in Zone 1 (42.4%) and lowest among districts in Zone 3 (27.8%). The percentage of districts with a policy addressing the use of radon-resistant new construction practices did not differ statistically across EPA radon zones (Table 1).

## 4. Discussion

The findings of this study show that school districts in EPA zones with the highest potential for elevated indoor radon levels were the most likely to have a policy requiring schools be tested for radon; yet, almost 60% of those districts lacked a radon testing policy. Regardless of zone, EPA recommends all schools test for radon because schools outside of high zones still can have elevated indoor radon levels [7].

Districts in zones with the highest potential for elevated indoor radon levels were no more likely to have a policy requiring the use of radon-resistant new construction practices than districts in zones with the lowest potential. Interestingly, the prevalence of policies addressing radon-resistant new construction practices was low among districts located in the zone with the highest predicted average indoor radon; only 37.5% of districts in Zone 1 had such a policy.

A number of resources exist that explain the importance of school radon testing and implementation of appropriate radon-related policies. Protocols for indoor radon testing in schools and radon-resistant new construction are available [8,9]. Additionally, radon programs exist in every state. These programs can help school districts develop testing programs and implement radon-resistant new school construction practices.

The findings of this study should be evaluated in the context of some limitations. First, district level data are not a direct measure of school practices, nor a measure of the enforcement of district policies. Second, these data are based on self-report. Finally, EPA radon zones are not a direct measure of indoor radon concentrations for any specific piece of property.

## 5. Conclusions

SHPPS data suggest many students and school staff would benefit from radon testing programs and implementation of radon-resistant new construction practices. Additionally, the findings of this study suggest a critical need for increased awareness about radon testing for schools and for radon-resistant new construction practices.

## Figures and Tables

**Table 1:** Percentage of school districts with policies for radon testing and radon-resistant new construction practices by the Environmental Protection Agency (EPA) radon zone.

EPA Radon Zone ^1^	Policy Requiring Schools Test for Radon-Percent (95% CI ^2^)	Policy Addressing Radon-Resistant New Construction-Percent (95% CI)
Zone 1 (>4 pCi/L)	42.4 (35.2, 49.9)	37.5 (30.8, 44.7)
Zone 2 (2–4 pCi/L)	37.7 (30.5, 45.5)	31.0 (25.3, 37.2)
Zone 3 (<2 pCi/L)	27.8 (21.1, 35.7)	30.5 (23.3, 38.7)
	χ^2^ = 3.9, *p* = 0.02	χ^2^ = 1.3, *p* = 0.28

^1^ Predicted average indoor radon; ^2^ Confidence interval.
